# Intracranial Grade II Meningioma Oligometastatic to the Cervical Spine

**DOI:** 10.7759/cureus.12809

**Published:** 2021-01-20

**Authors:** Jyotsna M Natarajan, Donald E Born, Griffith Harsh, Lawrence M Shuer, Scott G Soltys

**Affiliations:** 1 Department of Radiation Oncology, Stanford Cancer Institute, Stanford, USA; 2 Department of Pathology, Stanford University School of Medicine, Stanford, USA; 3 Neurological Surgery, University of California, Davis, Sacramento, USA; 4 Department of Neurosurgery, Stanford University Medical Center, Stanford, USA; 5 Department of Radiation Oncology, Stanford University School of Medicine, Stanford, USA

**Keywords:** atypical meningioma, metastases, oligometastasis, radiosurgery, spine

## Abstract

For intracranial meningiomas that metastasize extracranially, an oligometastatic state exists that is intermediate between incurable, widely metastatic disease and non-metastatic curable disease. Similar to oligometastatic cancer, aggressive local treatment of meningioma oligometastases is warranted, as it may be curable. We present a patient with multiply recurrent intracranial meningiomas over 19 years, with a transformation from grade I to grade II histology, with oligometastatic disease to the C5 vertebral body. Three years following definitive spinal stereotactic radiosurgery, she remains without evidence of other metastatic diseases. Our case highlights the oncologic concept that metastatic meningioma need not be widely disseminated and provides the clinical rationale for aggressive local treatment of an oligometastatic meningioma.

## Introduction

Meningiomas are the most common, non-malignant, central nervous system primary tumors [1]. World Health Organization (WHO) grade I meningiomas are considered benign, with a five-year recurrence rate of 7% to 25% after a gross-total resection [2]. WHO grade II and III meningiomas are known to be more aggressive in behavior, with recurrence rates between 25% and 52% and 50% and 94%, respectively [2].

Extracranial meningioma metastases occur in approximately 0.1% of all patients with intracranial meningiomas [3]. In case reports [3-8] and small series [2,9] of patients with extracranial spread of meningiomas, metastases typically involve multiple sites, most commonly the lung, liver, lymph nodes, and bone [10-11].

There is growing acceptance that the classical binary concept of metastatic progression, where patients are either widely metastatic and, therefore, incurable versus non-metastatic and curable, is outdated. An intermediate oligometastatic state exists when metastases are localized to a limited number of sites and can potentially be cured with local therapy [12]. Most studies have limited the number of metastases to either one to three or one to five sites to be considered oligometastatic [12-13], but these data are evolving. Local therapy, such as surgery or stereotactic radiosurgery (SRS), to oligometastatic disease can lead to survival rates comparable to patients with a non-metastatic disease [12].

We present a patient with multiply recurrent intracranial meningiomas over 19 years, with a transformation from grade I to grade II histology, who had an oligometastatic disease to the C5 vertebral body. Three years following spinal surgery and SRS, she remains without evidence of intracranial, spinal, or other metastatic diseases. Our case highlights the oncologic concept that metastatic meningioma need not be widely disseminated and provides the clinical rationale for aggressive local treatment of an oligometastatic meningioma.

## Case presentation

With institutional research board (IRB) approval, we retrospectively analyzed the treatment and outcomes of a patient who developed oligometastatic spinal metastasis from a multiple recurrent intracranial grade II meningioma.

Initial presentation and treatment: brain surgery #1 - year 0

A 34-year-old woman presented with headaches, weakness, and gait instability, which led to the diagnosis of a right frontoparietal parasagittal meningioma. She had a gross total resection, with the pathology of a WHO grade I meningioma.

Intracranial recurrence #1: brain surgery #2 - 18.3 years following initial diagnosis

**Figure 1:**
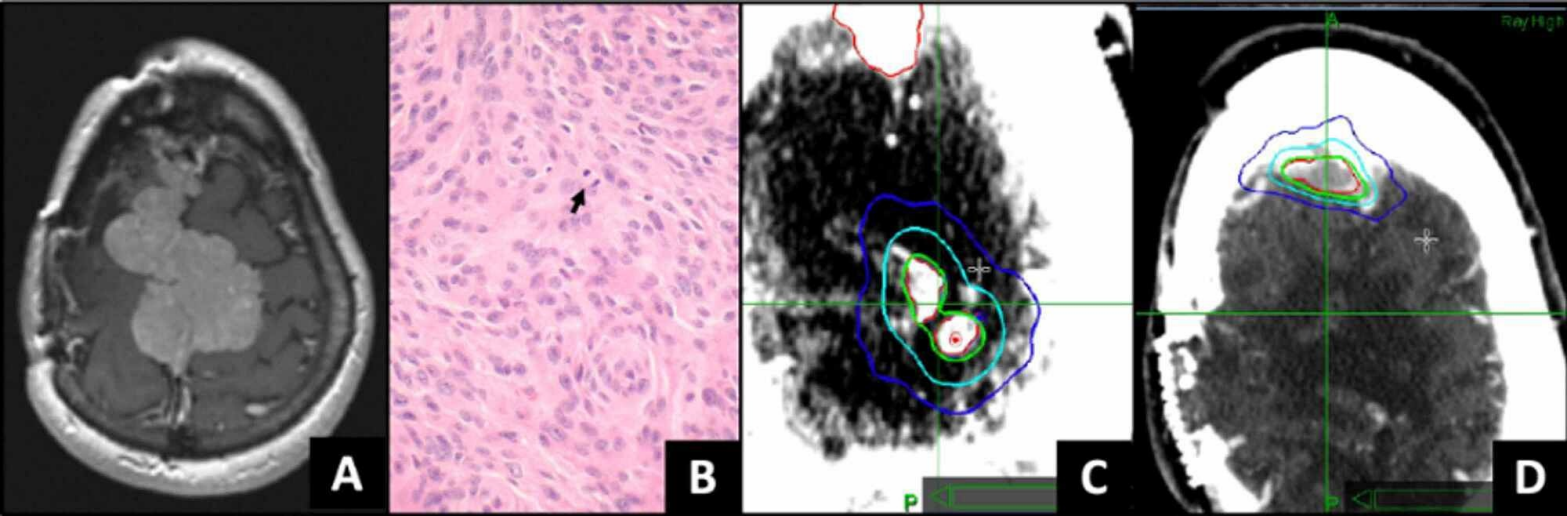
Intracranial Meningioma Treatment A 34-year-old woman had resection of a grade I falcine meningioma (imaging and pathology not available). The tumor recurred 18 years after the initial diagnosis (panel A). A gross total resection revealed transformation to grade II pathology (panel B – 40x magnification) with five mitotic figures (black arrow) per 10 high-power fields – a mitotic figure is noted by the black arrow). Stereotactic radiosurgery (SRS) targeted the recurrent tumor within (panel C – 22 years after initial diagnosis) and distant from (panel D – 26 years after initial diagnosis) the surgical sites of the recurrent tumor. In panels C and D, the SRS prescription isodose line of 18 Gy in one fraction is green, with the 50% dose line in cyan.

Following a seizure, the patient presented to the emergency department with imaging that revealed recurrent meningiomas, a 7 cm tumor at the vertex obstructing the superior sagittal sinus with an adjacent, separate 0.7 cm tumor (Figure 1A). These tumors were within and adjacent to the previous site of meningioma and not present on imaging six years prior. She had a bifrontal craniotomy with gross total resection of the tumors. Pathology revealed an atypical meningioma, WHO grade II, composed of a proliferation of cells with ovoid and spindle-shaped mildly pleomorphic nuclei, eosinophilic cytoplasm forming a syncytium, and arranged in poorly formed whorls. Characterization as atypical was based on the finding of up to five mitotic figures per 10 high-power fields (Figure 1B). She had no adjuvant radiotherapy.

Intracranial recurrence #2: SRS # 1 - 22.7 years following initial diagnosis

Four years later, surveillance magnetic resonance imaging (MRI) demonstrated recurrent disease at three sites (measuring 9 mm, 6 mm, and 4 mm) within the resection cavity of the recurrent grade II tumor (Figure 1C). These three tumors were treated with 16-18 Gy in one fraction with stereotactic radiosurgery (SRS) (Figure 1D). 

Intracranial recurrence #3: SRS # 2 - 26 years following initial diagnosis

Three years later, for progression at another area of dural thickening distant from the original sites of surgery, a 9 mm tumor was treated with SRS, 20 Gy in one fraction.

Extracranial progression: spine surgery #1 and spine SRS #1 - 26.4 years status post initial diagnosis

**Figure 2:**
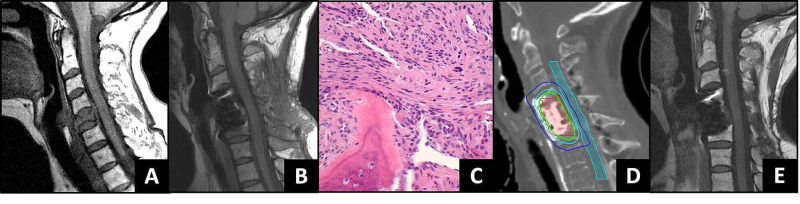
Spine Oligometastatic Treatment Twenty-six years after her initial diagnosis of a grade 1 intracranial meningioma and eight years following intracranial recurrence as a grade II, she presented with symptomatic C5 metastasis (panel A – sagittal T1 pre-contrast MRI with complete replacement of the vertebral body with pathologic fracture. There was a para-spinous extension into the soft tissue of the neck along the vertebral arteries). Following subtotal resection and stabilization (panel B – post-surgical T1 pre-contrast MRI), pathology revealed a grade II meningioma within the bone (panel C), concordant with the prior intracranial recurrence (in Figure 1B). She had definitive postoperative spinal stereotactic radiosurgery (SRS) to her residual tumor (panel D – spinal SRS plan. 27 Gy in three fractions (green isodose line) targeted the postoperative resection site (red contour)). The spinal cord (blue contour) was limited to a maximum of 21 Gy (cyan isodose line). Three years following spine SRS, she remains without evidence of spine (panel E), brain, or metastatic progression.

While being treated for the intracranial recurrence #3 above, in the setting of a three-month history of neck pain and stiffness, magnetic resonance imaging (MRI) showed a C5 compression fracture with a tumor in the epidural space encasing the right vertebral artery and extending into the neuroforamen (see Figure 2A), new since prior imaging. The tumor had no intradural extension to suggest that this was a more common intradural, extramedullary spinal meningioma or that this represented cerebral spinal fluid (CSF) spread of her intracranial tumors.

For stabilization and pathologic diagnosis, she underwent a C2-C6 laminectomy with C4-C6 anterior cervical fusion. Pathology revealed atypical meningioma, WHO grade II, within fragments of trabecular bone. The neoplastic proliferation, in addition to showing features typical of meningioma, had areas of sheet-like growth, necrosis, and macronuclei (see Figure 2C). Although mitotic figures were not as numerous, the histology was concordant with the prior intracranial recurrence #1 (Figure 1B). She was treated with postoperative spinal SRS, 27 Gy in three fractions, to the C4, C5, C6 vertebral bodies and the extraosseous extension along the right vertebral artery from C4 to C7 (Figure 2D).

Follow-up: 30.5 years following initial diagnosis

Three years following SRS of the spinal metastasis, she remains locally controlled in both her brain and spine. Surveillance body computed tomography (CT) imaging has displayed no evidence of other metastatic sites.

## Discussion

Meningiomas are the most common primary central nervous system tumor, accounting for approximately 38% of all brain tumors and 53% of all non-malignant brain tumors [1]. Approximately 25% of meningiomas are WHO grade II [1,14]. Following a gross total surgical resection, tumor local recurrence rates are between 7% and 25% for WHO grade I histology and up to 52% for WHO grade II meningiomas [2-3,15].

Despite a high local recurrence rate for grade II meningiomas, extracranial metastases are extremely rare, occurring in approximately 0.1% of patients [16]. Multiple case series of metastatic meningioma (see Table 1), including the largest recent report of six patients by Kessler et al., suggest that once a meningioma has hematogenously spread to develop metastases, it can behave similarly to hematogenously disseminated cancer, with metastatic involvement of multiple extracranial sites, including the spine, bones, liver, and lungs. In the selected series in Table 1, 77% of patients with extracranial spread of meningioma had the involvement of more than a solitary site at the time of diagnosis of metastatic disease.

**Table 1:** Reported cases of extracranial metastases of WHO grade II atypical meningiomas Adapted from Kessler et al. [2], Ward et al. [3]

Citation	Number of patients reported	Location of original tumor	Time from original diagnosis to extracranial metastasis	Tumor grade	Number of metastatic sites	Location of metastatic sites	Length of follow-up after time of metastases	Status
Abboud 2009 [10]	1	Left tentorium	13 years	II	2	Left and right sacroiliac and gluteus muscle regions	Not reported	Alive without disease progression
Kessler 2017 [2]	1 of 4	Falcine	14 years	I, transformed to II when metastasized	1	Lung	Hospice after 1 year	Deceased
Kessler 2017 [2]	2 of 4	L parietal, R frontoparietal lobes	23 years	Originally II, transformed to III intracranially before metastasis	2	Lung, Mediastinum	Hospice after 4 years	Deceased
Kessler 2017 [2]	3 of 4	L frontal, L hemisphere extending to calvarium	6 years	Originally II, transformed to III intracranially before metastasis	2	Liver, Spine	11 months	Deceased
Kessler 2017 [2]	4 of 4	Superior sagittal sinus	7 years	II	1	Liver	Hospice after 3 weeks	Deceased
Lee 2009 [4]	1	Right lateral ventricular	14 months	II, transformed into III	8	T5, T7, T10, L1, L3, L4, S1, and S2 vertebrae of the spine	1 month	Deceased after 1 month
Olson 1994 [5]	1	Left frontal lobe	17 months	II	4	T6, T11, pulmonary and hilar nodes	N/A	N/A
Paix 2017 [6]	1	Right parietal lobe	3 months	II	8	T8, T10, L1, L4, 8^th^ rib, coccyx, lungs, and liver	Not Reported	Alive with metastases
Palmer 1994 [7]	1	Right posterior fossa	12 years	II	2	L5 extending into the right pedicle, Lung	Hospice for an undisclosed period	Deceased after 6 months
Pinsker 2005 [8]	1	Right frontal lobe	5 years	II	2	C3 and C4 vertebral bodies	Not Reported	Alive with metastases
Singh 2016 [9]	1 of 2	Left middle cranial fossa	3 years 4 months	II, transformed to III	4	L4, T7, T8, T9, T10, S2 to left upper sacral foramina, spinal cord	5 months	Deceased 5 months after finding metastases
Singh 2016 [9]	2 of 2	Right frontal lobe	6 years	I, transformed into II	9	Multiple vertebrae, distal left clavicle, proximal left humerus	1 year	Deceased 1 year after finding metastases
Current Case	1	Right frontal parasagittal lobe	26 years	I, transformed into II prior to extracranial metastasis	1	C5 vertebral body	3 years	Alive, with no evidence of disease

Historically, the state of a patient’s cancer has been categorized into two groups: locally confined and potentially curable or widely metastatic and incurable. In 1995, Hellman and Weichselbaum proposed an intermediate state between locally confined and widely metastatic [17]. This oligometastatic state, where a limited number of metastatic sites are present prior to developing widely disseminated metastases, may be curable with definitive local treatment of each oligometastasis [12-13]. Consensus recommendations and a proposed classification of the various oligometastatic states were recently published [18].

There are no clear risk factors associated with why some patients may develop disseminated metastatic meningiomas compared to patients with oligometastatic disease as in our case example. Retrospective [19] and prospective [20] data find that recurrent meningiomas behave more aggressively than newly diagnosed meningiomas of the same grade. One may hypothesize that the more aggressive behavior of locally recurrent tumors increases the risk of hematogenous dissemination; all of the previously reported cases of metastatic meningioma [2] occurred following a local recurrence of the primary tumor.

Despite multiple intracranial recurrences in our patient, the transformation to a higher grade, and subsequent metastasis, one may still hypothesize that the very long disease-free intervals between recurrences connote less aggressive behavior than recurrent tumors seen in other patients. The outcome of our patient is not necessarily notable for durable local control of her metastatic site with spinal radiosurgery; ablative radiotherapy is a standard of care treatment for oligometastatic disease. Unusual is her oligometastatic presentation, with no further evidence of recurrence in three years of follow-up. Of note, she did not receive any systemic agents that could decrease the risk of further metastatic disease.

The oligometastatic state exists for cancer that has metastasized. We believe that our patient demonstrates that the oligometastatic state can apply to metastatic meningioma. As seen in Table 1, once metastatic, at least 77% of patients have widespread metastases at multiple sites. Our patient had a solitary extracranial metastasis to the C5 vertebral body, treated with surgical stabilization and definitive spinal radiosurgery. Three years following her initial metastatic disease, she has local control within the brain and spine, with no further extracranial metastases.

## Conclusions

The oligometastatic state exists for patients with metastatic meningioma. As with metastatic cancer, the oligometastatic state is potentially curable, with definitive local treatment of metastatic sites. Our case report serves to give hope to patients with oligometastatic extracranial spread of intracranial meningiomas.
